# Overexpression of the *Liriodendron tulipifera BOP2* Gene (*LtuBOP2*) Affects Leaf Margin Development in Transgenic *Arabidopsis thaliana*

**DOI:** 10.3390/ijms24043262

**Published:** 2023-02-07

**Authors:** Yanqing Zhao, Lingmin Wei, Shaoying Wen, Huogen Li

**Affiliations:** 1 State Key Laboratory of Tree Genetics and Breeding, Co-Innovation Center for Sustainable Forestry in Southern China, Nanjing Forestry University, Nanjing 210037, China; 2 College of Forestry, Nanjing Forestry University, Nanjing 210037, China

**Keywords:** *Liriodendron tulipifera*, *BOP2*, proximal–distal, serrated leaf, *Arabidopsis thaliana*

## Abstract

BLADE-ON-PETIOLE 2 (*BOP2*) plays a pivotal role in leaf morphogenesis. *Liriodendron tulipifera* is a suitable model for exploring the molecular mechanisms underlying leaf serration formation, which are largely unknown. Here, we isolated the full-length *LtuBOP2* gene and its promoter from *L. tulipifera* and characterized its function in leaf morphogenesis through multidimensional approaches. The spatiotemporal expression pattern of *LtuBOP2* indicated the high expression of *LtuBOP2* in stems and leaf buds. We constructed *LtuBOP2* promoter, fused the promoter sequences to the β-glucuronidase (*GUS*) gene, and then transformed them into *Arabidopsis thaliana*. Histochemical GUS staining results indicated that *GUS* activity was higher in petioles and the main vein. *LtuBOP2* overexpression in *A. thaliana* caused moderate serration in the leaf tip, owing to the increased number of abnormal lamina epidermal cells and defective vascular tissue, thus indicating a novel role of *BOP2*. The ectopic expression of *LtuBOP2* in *A. thaliana* promoted the expression of the lateral organ boundary gene ASYMMETRIC LEAVES2 (*AS2*) and inhibited JAGGED *(JAG)* and CUP-SHAPED COTYLEDON2 (*CUC2*) expression to establish leaf proximal–distal polarity. Moreover, *LtuBOP2* participated in leaf serration formation by promoting the antagonistic relationship between *KNOX I* and hormones during leaf margin development. Our findings revealed the role of *LtuBOP2* in the proximal–distal polarity formation and development of leaf margin morphology, providing new insights into the regulatory mechanisms of the leaf formation development of *L. tulipifera*.

## 1. Introduction

The growth and propagation of plants are always inseparable from energy supply; since energy production requires the assistance of photoreception, respiration, and transpiration in the leaves, leaves are considered the main source organs for higher plants to produce energy by photosynthesis [1,2]. Accordingly, the leaf size and shape influence the growth condition and developmental quality of plants [3]. During the evolutionary process of leaves, the changes in leaf size and shape can enhance their adaptability to natural environments. For example, plants with leaf serrations can improve their adaptability to stressful conditions, such as drought, heat, and wind, by adjusting their irradiation area. Furthermore, serrated leaves can improve the light transmittance of the tree crown and have advantage in light interception for lower layer leaves in shade conditions [4,5]. Therefore, leaves are an ideal system to explore plant organ morphogenesis.

Leaf development is an iterative process of organ initiation from the meristem, which can be divided into three stages: primordium initiation (PI), primary morphogenesis (PM), and secondary morphogenesis (SM) [6]. First, the stem cells originate as the main lateral appendage of the stem in the peripheral zone (PZ) of the shoot apical meristem (SAM) [7], where cells divide rapidly and can differentiate at specific sites to form flower primordium, lateral branch primordium, and leaf primordium [8]. Further, during PM, a boundary region separates the proximal region of the lateral organ from SAM [9]. Leaves with vigorous differentiation potential grow along the three-dimensional axis (proximal–distal, adaxial–abaxial, and medio–lateral axes), thereby establishing of leaf polarity [10,11]. The cells divide and differentiate continuously to form adaxial–abaxial axis, developing from a mass cell to a flat structure [12,13]. Meanwhile, in the PM stage, local cells at the leaf margin proliferate constantly, then serrations occur at the mature leaf margin. [14]. Finally, the leaf undergoes cell expansion, division, and differentiation to expand the leaf area and develop stomata and epidermal hairs along the polarity axis, concluding the leaf shape development [15].

Determination of the leaf shape involves a complex genetic network and hormones [16,17]. *KNOX I* and boundary genes are reportedly involved in leaf development. The *KNOX I* family genes include SHOOT MERISTEMLESS (*STM*), BREVIPEDICELLUS (*BP* or *KNAT1*), KNOTTED IN *Arabidopsis thaliana* 2 (*KNAT2*), and *KNAT6*. Initiation of leaf founder cells requires repressed expression of *KNOX I*; ASYMMETRIC LEAVES1 (*AS1*) transcription factor plays an essential role in repressing *KNOX I* expression [18]. Once cells begin to divide and differentiate, the new leaf primordium separates from the SAM to form a boundary region, and the involved genes are collectively called boundary genes. These boundary genes include ASYMMETRIC LEAVES2, BLADE-ON-PETIOLE 1/2 (*BOP1/2*), JAGGED (*JAG*), JAGGED LATERAL ORGANS *(JLO*), *A. thaliana* HOMEOBOX1 (*ATH1*), and CUP-SHAPED COTYLEDON (*CUC*). When the leaf primordium originates at the PZ, *CUC* is specifically expressed in boundary positions and interacts with the KNOX I protein to promote boundary formation [19]. *CUC* expression is influenced by auxin maxima and determined by the expression pattern of PINFORMED1 (*PIN1*). The PIN1-CUC regulation pathway performs important roles in organ boundary establishment, determining the position and size of serration [20]. *JAG* overexpression results in a smaller leaf and a phenotype with leaf margin serration [21]. As a transcription inhibitor, *JAG* controls the distal axis development of leaves by regulating cell division and is itself regulated by *BOP* [22]. In *A. thaliana* leaf formation, *BOP* regulates cell activity in lateral organs and polarity establishment in proximal–distal axis [23]. BOP1/2 proteins can dimerize in vivo and associate directly with the *AS2* promoter, thereby regulating leaf morphogenesis by repressing *KNOX I* and *JAG* transcription [24]. Conserved mechanisms for *BOP-*mediated leaf morphogenesis with patterning along the proximal–distal axis have now been identified in several species. Some examples include NOOT in *Medicago truncatula* and COCHLEATA/COCH in *Pisum sativum* (*A. thaliana BOP* orthologs genes), whose mutants have serrated phenotypes similar to *bop* [25]; *SlBOP2* regulates leaf shape development in tomato (*Solanum lycopersicum*) by repressing *KNOX I* expression [26], suggesting a conserved function of *BOP* in leaf organ development. In summary, although the molecular mechanism between *BOP* and *KNOX I* has been reported in detail, it remains unclear whether there is a regulatory relationship between *BOP* and other genes involved in leaf development.

*Liriodendron,* a genus of the Magnoliaceae family, is a tertiary relict plant, of which only two species remain—*L. chinense* and *L. tulipifera*. *Liriodendron* plants are commonly used as ornamental trees, owing to their peculiar leaf shapes and tulip-like flowers. In recent years, with the advent of next-generation sequencing and release of the *Liriodendron* genome, research on leaf morphogenesis and development in *Liriodendron* plants has been increasing [27]. Yang and co-authors reported that nine *NAC* genes were involved in leaf senescence regulation [28]. Ma and co-authors defined four developmental stages of young leaves in *Liriodendron* based on the results from using paraffin sections and scanning electron microscopy (SEM); they determined that the leaf petiole develops in the P2 phase, indicating the establishment of proximal–distal polarity, and that morphological features such as leaf veins and serration could be observed in the P3 phase [29]. Further studies revealed that *LcKNOX6*, homologous to *AtKNAT6* in *A. thaliana*, negatively regulates *AtKNAT1*, *AtGA20ox1*, *AtPIN1,* and *AtAS2* expression, which causes the emergence of leaf lobes and ultimately affects leaf shape development [30]. Li et al. verified that *LcNAM* was expressed in all parts of the leaf, and *LcCUC1/2/3* was a specific expression factor for the leaf margin. Functional analysis via genetic transformation illustrated that *CUC1* contributes to the formation of the crinkled leaves phenotype, and *CUC2* increases the number of leaves and causes different degrees of serration [31]. Based on these results, Wen et al. elucidated the mechanism by which *LcCUC2* affects leaf lobes, showing that the ectopic expression of *LcCUC2* caused the leaf petiole cotyledons to become longer and narrower, upregulated the *KNAT2/6* expression, and ultimately influenced the development of leaf margin serrations by regulating auxin content [32]. Wei et al. reported that *LtuJAG* overexpression altered leaf margin shape in *A. thaliana*, resulting in leaves with serration and asymmetric differentiation and growth in the medio–lateral and proximal–distal positions [22]. Briefly, although these studies have uncovered some aspects of the mechanisms underlying leaf shape development in *Liriodendron*, the precise molecular mechanism remains unclear. Moreover, growth and developmental processes in plants are controlled by the expression of genes, while gene transcription levels are influenced by promoters containing cis-acting elements [33]. In this study, to reveal the potential function of *LtuBOP2* in the leaf margin development of *Liriodendron* plants, we isolated *LtuBOP2* and its promoter from *L. tulipifera* and then ectopically expressed it in *A. thaliana* wild-type and *bop2* mutants. Consequently, we obtained a 35S::*LtuBOP2* transgenic plant with smaller and lobed leaves and confirmed the regulatory relationship between *LtuBOP2* and other leaf development genes. Our results provided a potential genetic mechanism of leaf margin development, as well as insights for breeding ornamental cultivars in *L. tulipifera*.

## 2. Results

### 2.1. Cloning and Bioinformatics Analysis of LtuBOP2

**Figure 1:**
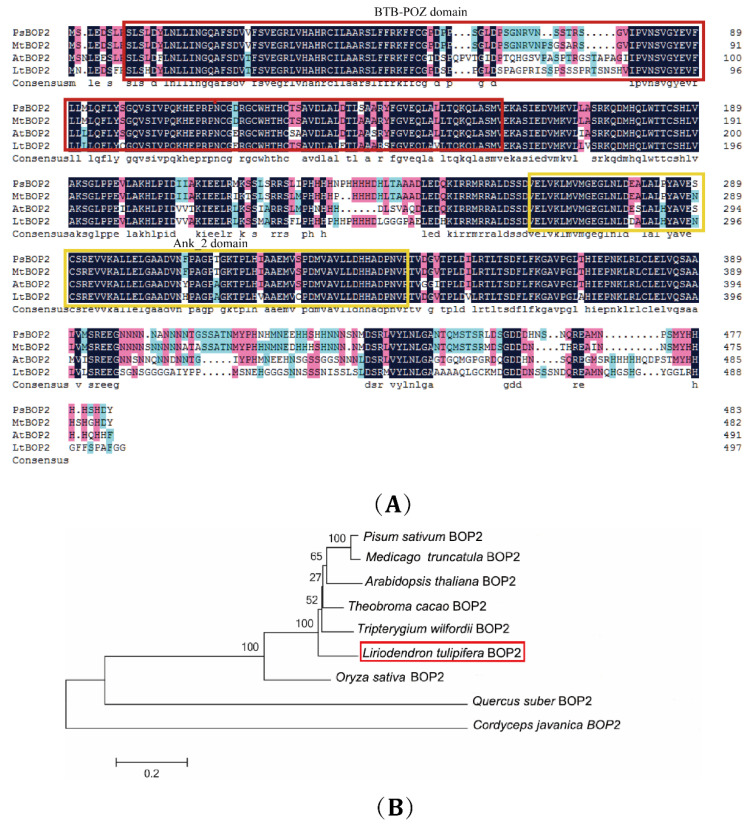
Sequence and phylogenetic analysis of LtuBOP2 protein. (**A**) Multi-sequence alignment of the predicted amino acid sequence of LtuBOP2 with PsBOP2, MtBOP2*,* and AtBOP2 amino acid sequences. Multiple sequence alignment was performed using the DNAMAN software. Identical residues among all four proteins are shaded in black. Pink and light blue shades indicate identical residues that appeared in three and two sequences, respectively. The red box indicates the BTB-POZ domain, and the yellow box indicates the ankyrin-repeats Ank_2. Lt: *Liriodendron tulipifera*, Ps: *Pisum satioum* (XP_050911308.1), Mt: *Medicago truncatula* (XP_003625012.1), At: *A. thaliana* (Q9ZVC2.1). (**B**) An unrooted phylogenetic tree of the LtuBOP2 amino acid and BOP2 amino acids in different species. LtuBOP2 is indicated by the red box. The phylogenetic tree was reconstructed using the MEGA7 software and the Neighbor-Joining (NJ) method based on 1000 bootstrap replicates. Bootstrap support values are indicated. *Pisum sativum* (AET34792.1), *Medicago truncatula* (XP_003625012.1), *A. thaliana* (Q9ZVC2.1), *Theobroma cacao* (EOY09068.1), *Tripterygium wilfordii* (KAF5737654.1), *Oryza sativa* (Q2RAQ5.1), *Quercus suber* (XP_023898361.1), *Cordyceps javanica* (TQW11789.1).

The BOP2 protein was analyzed using the NCBI Conserved Domain software and obtained as a member of the BTB family with BTB-POZ (Poxvirus Zinc Finger Protein) domain and ankyrin-repeats (Ank_2) domain (Figure 1). BOP proteins are members of the small NON-EXPRESSOR OF PATHOGENESIS-RELATED GENES (NPR) family, characterized by a BTB-POZ domain at the N-terminus and ankyrin-repeats (ANK) located near the C-terminus. *LtuBOP2* has sequence similarity with the *BOP2* of other species in protein alignment. Multiple alignment results showed that these sequences were highly homologous, and all contained conserved N-terminal and polymorphic C-terminal sequences. *L. tulipifera* BOP2 has the closest genetic relationship with *Tripterygium wilfordii* BOP2 and is relatively close to *Theobroma cacao* BOP2. However, it is relatively far from *Pisum sativum* BOP2, *Medicago truncatula* BOP2, and *A. thaliana* BOP2, which are more highly evolved, indicating that this amino acid sequence is highly conserved in the BTB domain and belongs to the BTB family, named *LtuBOP2*.

### 2.2. Spatiotemporal Expression Pattern of LtuBOP2

**Figure 2:**
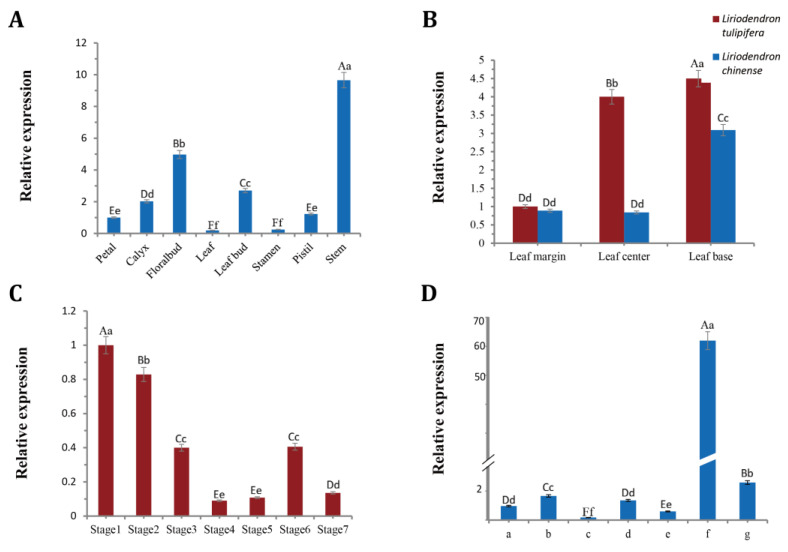
*LtuBOP2* expression pattern was analyzed using quantitative RT-qPCR. *LcACTIN97* was used as a reference gene. (**A**) Expression pattern of *BOP2* in eight different tissues of *L. tulipifera*; (**B**) Expression pattern of *BOP2* in the leaf base, leaf center and leaf margin of *L. tulipifera* and *L. chinense*, respectively; (**C**) Expression pattern of *BOP2* in developmental stage of leaf in *L. tulipifera*. Stage1–Stage2: leaf bud germination stage; Stage3: young leaf stage; Stage4–Stage6: mature stage; Stage7: senescence stage; (**D**) Expression pattern of *BOP2* in different parts of leaf of *L. tulipifera.* a, c, and e: leaf tooth protrudent, b and d: leaf tooth sinus, g: leaf middle, f: petiole. The one-way ANOVA Tukey’s test was applied for statistical analysis. The error bars indicated the standard deviation between the three biological repeats. The capital letters indicate significant differences at *p* < 0.01 level, the lowercase letters indicate significant differences at *p* < 0.05 level.

As shown in Figure 2A, the quantitative analysis results revealed that the *BOP2* gene had different expression levels in the eight tissues of *L. tulipifera*. The expression in stem was significantly the highest among the other tissues. The expression in the flower bud and leaf bud was higher than that in the other tissues (excluding the stem), and a lower expression was observed in the petals, calyx, stamens, pistils, and leaves. Leaf buds and flower buds originated from leaf primordium and flower primordium, respectively, and both contained the shoot apical meristem, while the stems contained the lateral meristem, suggesting that *LtuBOP2* is involved in meristem development.

In *L. tulipifera*, *BOP2* expression was the highest in the leaf base, followed by leaf middle, and lowest in the leaf margin (Figure 2B). In *L. chinense*, *BOP2* expression was also highest in the leaf base, followed by leaf margin, and lowest in leaf middle. Collectively, these results indicate that *BOP2* was mainly expressed in the leaf base in *L. tulipifera* and *L. chinense*.

The time-specific expression condition of *LtuBOP2* was surveyed at the leaf bud growth stage (stages 1 and 2), young leaf stage (stage 3), mature stage (stages 4 to 6), and senescence stage (stage 7) (Figure 2C). The *LtuBOP2* expression decreases in correspondence with the expansion of leaves in the growth stage of leaf bud, and this expression is higher than that in the mature stage. This suggests that *BOP2* may be expressed mainly during leaf bud differentiation to control organ development.

To further detect the leaf spatial-specific expression of *LtuBOP2*, we subjected the tooth tips (a, c, and e), tooth sinuses (b and d), petiole (f), and middle part (g) of *L. tulipifera* leaves to quantitative reverse transcription-PCR (RT-qPCR). The *LtuBOP2* transcript level was visibly highest in the leaf petiole and substantially higher in the leaf tooth sinuses (b and d) than in the leaf tooth tips (a, c, and e) (Figure 2D). These results indicate that *LtuBOP2* may be involved in the formation of petiole development.

### 2.3. pLtuBOP2 Sequence Analysis

To determine tissue specificity, the *LtuBOP2* promoter fragment (*pLtuBOP2*), situated approximately 2 Kb upstream of *BOP2* in *L. tulipifera*, was separated and analyzed for potential regulatory elements (Supplementary Materials Table S2), including light-responsive cis-elements, G-box, GT1-motif, ACE, AE-box, GTGGC-motif, and chs-Unit 1 m1. The abiotic stress response elements included STRE (thermal induction), LTR (low temperature), and as-1 (cold). Moreover, a few hormone-responsive elements were identified, including ABRE (ABA), P-box (GA), CGTCA-motif (Methyl Jasmonate), TATC-box (GA), and TGACG-motif (Methyl Jasmonate). In addition, *pLtuBOP2* specifically contained meristem expression regulatory elements, i.e., the CAT-box. The sequence analysis of the *LtuBOP2* promoter region suggested that *LtuBOP2* may be involved in regulating a range of processes, including abiotic stresses, responses to hormones, and meristem development.

**Figure 3:**
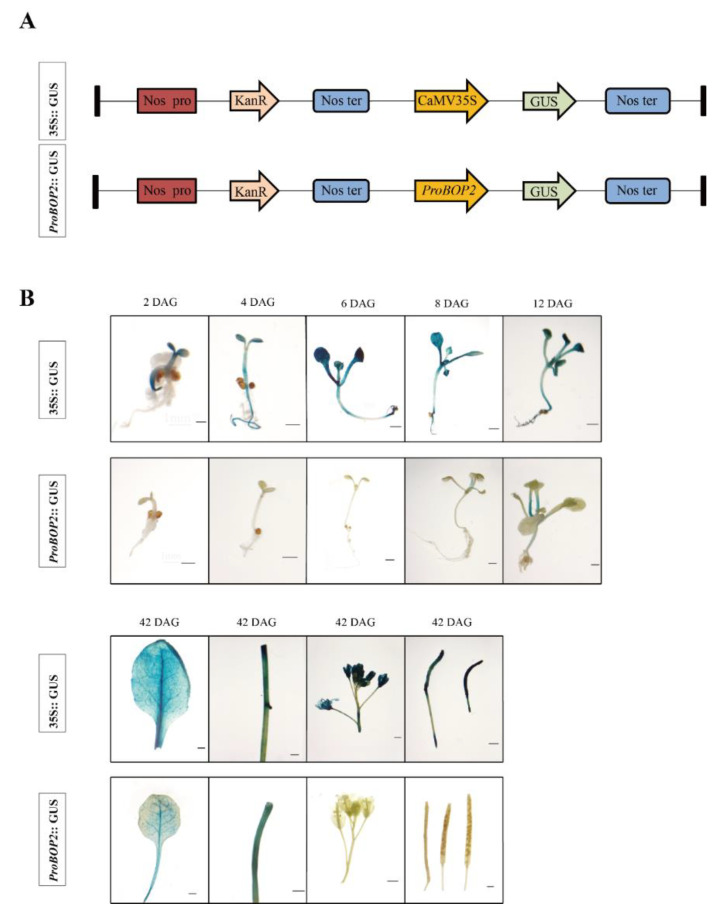
GUS histochemical staining in transgenic *Arabidopsis* T2 overexpression lines. (**A**) The T-DNA construction diagram of *Arabidopsis* transformation. Nos: nopaline synthase gene; KanR: Kanamycin resistance gene; GUS: β-glucuronidase gene; (**B**) Tissue staining showing expression pattern of *pLtuBOP2*::GUS and 35::GUS in seedlings (two leaves stage, four leaves stage, six leaves stage), cotyledons, rosette leaves, stems, inflorescences, siliques, and seeds sampled form transgenic *Arabidopsis* seedlings. DAG: day after germination. Scale bar: 1 mm.

Figure 3B shows *GUS* gene expression driven by *pLtuBOP2* at the two-leaf, four-leaf, six-leaf, and flowering stages of *A. thaliana. GUS* gene expression driven by *pLtuBOP2* was not detected in 2d, 4d, and 6d seedlings. After leaf bud differentiation at 8–12d, GUS activity was strongest in the newly differentiated petiole, but it was almost nonexistent in the radicle, hypocotyl, and leaf. The *LtuBOP2* promoter drove GUS expression in leaves and stems but not in inflorescences and pods. In addition, we observed that *LtuBOP2* promoter activity was highest in petiole and the main vein, followed by the secondary vein. Among different tissues, *pLtuBOP2* activity was found to be the highest in the stem, indicating that *LtuBOP2* plays a role in the apical meristem at the early stage of leaf bud differentiation; further, it was strongly expressed in the vascular bundle of mature tissues.

### 2.4. Phenotypic Analysis of LtuBOP2 Transgenic Plants

**Figure 4:**
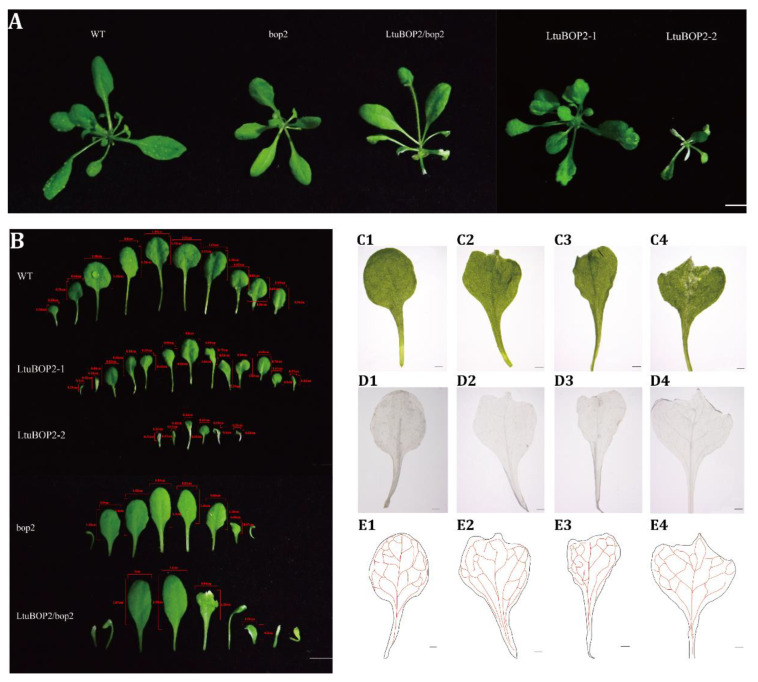
Phenotype of 35S::*LtuBOP2* transgenic *A. thaliana.* (**A**) *A. thaliana* wild-type (WT), *A. thaliana BOP2* mutant (*bop2*), over-expressing *LtuBOP2* in *bop2* (35S::*LtuBOP2*/*bop2*); two lines of over-expressing *LtuBOP2* in WT (35S::*LtuBOP2*-1 and 35S::*LtuBOP2*-2); (**B**) The leaves of WT, 35S::*LtuBOP2* transgenic *A. thaliana*, *bop2* and 35S::*LtuBOP2*/*bop2*; (**C**–**E**) C1, D1 and E1 represent *bop2*, C2, C3, D2, D3, E2 and E3 represent 35S::*LtuBOP2*, C4, D4 and E4 represent 35S::*LtuBOP2/bop2*. (**D**) Leaves were decolourized with 75% (V/V) ethanol. (**E**) Illustrations of the vascular patterns. (**A**,**B**) scale bar = 1 cm. (**C**–**E**) scale bar = 1 mm.

To monitor the effect of *LtuBOP2* on plant leaf morphogenesis, we transformed the *LtuBOP2* into *A. thaliana* wild-type and *bop2* mutants. To facilitate the further investigation of the leaf shape, we used a stereoscopic microscope to observe the leaf structure and found that 35S::*LtuBOP2*-1 and 35S::*LtuBOP2*-2 had shorter leaf lengths at the proximal–distal axis than the wild-type and 35S::*LtuBOP2*/*bop2* (Figure 4). Moreover, the leaf base morphology was unchanged, and there was a clear serration at the tip of the plant leaves. These results suggest that *BOP2* affects the establishment of leaf proximal–distal polarity.

The network system connecting the leaf veins determines the leaf shape and size, and the leaf vein formation is related to polarity establishment. An observation of leaf veins with the serrated phenotype revealed a reduction in the number of lateral veins and slender veins differentiated in 35S::*LtuBOP2* and 35S::*LtuBOP2*/*bop2*. *LtuBOP2* may have altered the vascular development of leaves to affect leaf shape, and leaf tip serration may be attributed to the incomplete differentiation of the plant’s distal axis.

### 2.5. Leaf Morphology of Transgenic A. thaliana Lines

**Figure 5:**
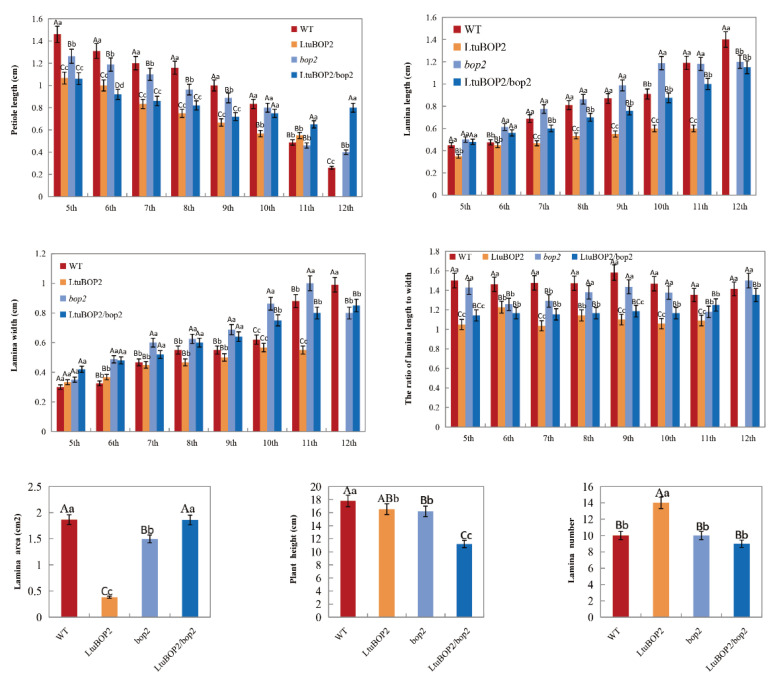
The effect of the *LtuBOP2* on leaf size in *A. thaliana.* Numerical changes in petiole length, leaf length, leaf width, leaf area, leaf number, plant height and aspect ratio for WT, 35S::*LtuBOP2*, *bop2* and 35S::*LtuBOP2*/*bop2* in 32 d *A. thaliana*. The error bars represent the standard deviation among the three biological repeats. The 5 th, 6 th, 7 th, 8 th, 9 th, 10 th, 11 th and 12 th represent the fifth leaf, the sixth leaf, the seventh leaf, the eighth leaf, the ninth leaf, the tenth leaf, the eleventh leaf and the twelfth leaf of *A. thaliana*. The one-way ANOVA Tukey’s test was applied for statistical analysis. Values are means ± SD (n ≥ 30). The capital letters indicate significant differences at *p* < 0.01 level, the lowercase letters indicate significant differences at *p* < 0.05 level.

To investigate the effect of *LtuBOP2* ectopic expression on leaf development in *A. thaliana*, we measured petiole length, lamina length, lamina width, lamina area, number of leaf, plant height, and the ratio of lamina length to width at 32 d after germination (Figure 5). The 35S::*LtuBOP2* plants were shorter and smaller than the wild-type in terms of petiole length, leaf length, leaf width, and leaf area; 35S::*LtuBOP2*/*bop2* plants were also shorter and smaller in all aspects than *bop2* plants, which suggested that *BOP2* overexpression inhibited normal leaf development. *LtuBOP2* expression in *bop2* mutants was not very different from that observed in the wild-type. Although the leaf-width values of 35S::*LtuBOP2*/*bop2* were initially larger than those of the wild-type and mutant, other aspects such as petiole length, leaf length, and aspect ratio were smaller. The loss and restoration of *BOP2* had little effect on leaf shape size; thus, *BOP2* had a certain inhibitory effect on the leaf size and shape development. However, *BOP2* alone was not enough to cause large changes in leaf shape and may coordinate with other genes to jointly control leaf development.

### 2.6. SEM Observation of Transgenic A. thaliana Lines

**Figure 6:**
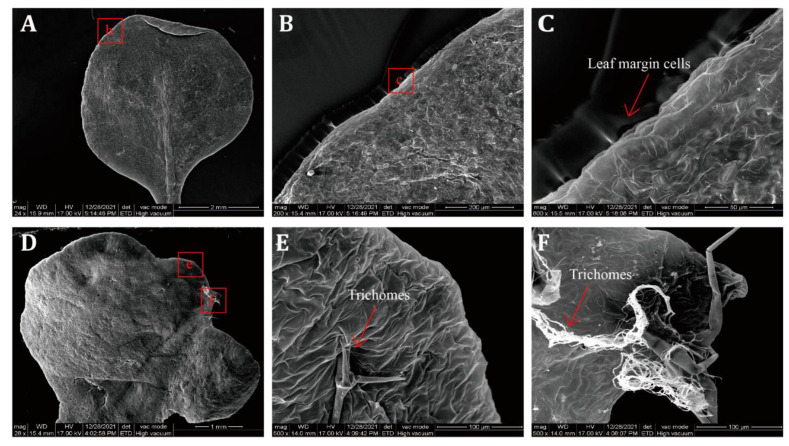
The SEM imaging comparison of leaf margin of 35S::*LtuBOP2* transgenic *A. thaliana* and WT*. (***A**–**C**): after the germination of 32 d rosette leaves of wild-type *A. thaliana,* where (**B**) is the local magnification of (**A**), and (**C**) is the local magnification of (**B**). The red box of b in (**A**) and the red box of c in (**B**) represent the location of (**B**,**C**). (**D**–**F**): after the germination of 32 d rosette leaves of 35S::*LtuBOP2* transgenic *A. thaliana*, where (**E**,**F**) are the local magnifications of (**D**). The red box of e and f in (**D**) represent the location of (**E**,**F**)

To better explore the mechanism of leaf margin serration formation, cells in the leaf epidermal and margin lobe sites of 35S::*LtuBOP2* and wild-type *A. thaliana* were observed using SEM. Leaf epidermal cells of the wild-type were irregular in shape, with small size differences. They were essentially closely arranged in an inlaid manner. Palisade cells on the leaf margins were arranged tightly and neatly in an elongated shape (Figure 6A–C). The leaf margin of *A. thaliana* is defined by elongated boundary cells. Compared with the relatively smooth leaf margin of the wild-type *A. thaliana*, the leaf margin boundary cells of the 35S::*LtuBOP2* lines disappeared, followed by the leaf margin formation via an arrangement of exuberantly dividing mesophyll cells. Notably, the epidermal cells at the leaf tooth margins proliferate abnormally, with many filamentous appendages derived from the roots of the epidermal hairs (Figure 6D–F). These results indicated that *LtuBOP2* affected cell proliferation and expansion in the early stages of leaf serration development, and played a prominent role in promoting cell division.

### 2.7. LtuBOP2 Affects the Expression of Genes Related to Leaf Shape Development

**Figure 7:**
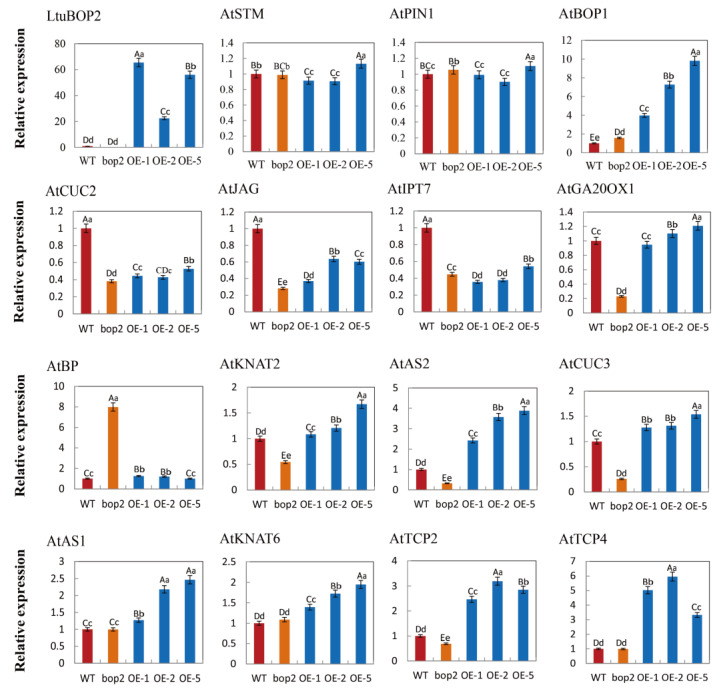
RT-qPCR was used to analyze the expression of genes in the 10-day-old wild-type plants (WT), three independent *LtuBOP2* overexpression lines (OE1, OE2, and OE5), and *bop2*. *AtACTIN2* served as the reference gene. The gene expression of WT as a control, the error bars indicated the standard deviation between the three biological repeats. The one-way ANOVA Tukey’s test was applied for statistical analysis. The error bars indicated the standard deviation between the three biological repeats. The capital letters indicate significant differences at *p* < 0.01 level, the lowercase letters indicate significant differences at *p* < 0.05 level.

Upon analyzing the plant appearance, an incision phenotype at the leaf tips was found in the *LtuBOP2* transgenic strain. To explore how the *LtuBOP2* gene regulates leaf shape development in *A. thaliana*, we first used the NCBI online database to identify the reported associated genes, mainly the genes associated with *BOP2* gene regulation from previous studies. The expression of these genes in *A. thaliana* seedlings overexpressing *LtuBOP2* was detected using RT-qPCR. As shown in Figure 7, we obtained three transgenic lines (OE1, OE2, and OE5) that overexpressed *LtuBOP2* at the T3 generation. There was little change in *AtSTM* and *AtPIN1* expression in the *bop2* and overexpression lines compared to that in the wild-type. *AtBOP1* was upregulated both in *bop2* and *LtuBOP2*-OE lines, demonstrating that *BOP1* is redundant with *BOP2*. In contrast, *AtCUC2*, *AtJAG*, and *AtIPT7* were downregulated in *bop* and *LtuBOP2*-OE lines, indicating that *BOP* may not act through one pathway but in combination with other genes to co-repress their expression. In *LtuBOP2*-overexpressing plants, *AtBP* expression was slightly higher than that in the wild-type, but *AtBP* expression was significantly increased in the *bop2* mutants, indicating that *BOP2* may have an inhibitory effect on *BP*. Furthermore, *AtKNAT2*, *AtAS2, AtGA20ox1,* and *AtCUC3* were upregulated in the *LtuBOP2*-OE and downregulated in the *bop2* lines, demonstrating that *BOP2* is positively correlated with the expression of these genes. In addition, *AtAS1*, *AtKNAT6,* and *AtTCP2/4* expression did not change in *bop2* lines compared to that in the wild-type but was increased in the *LtuBOP2*-OE lines, indicating that *BOP2* may affect their expression indirectly rather than directly. In conclusion, these results indicate that there may be multiple pathways for *LtuBOP2* to regulate different genes related to leaf shape development, thus affecting the morphological development of plant leaf margin from different aspects.

## 3. Discussion

### 3.1. Function of Domain in the LtuBOP2 Gene

While our previous studies provided preliminary definitions for the developmental stage and morphological characteristics and identified candidate genes in relation to the leaf development of *L. chinense* based on anatomical observations and transcriptome assays [28,29], little is known about the regulatory pathway or molecular mechanisms related to leaf shape development. Therefore, we compared the expression of some genes in the database of Ma et al. and identified the key gene *BOP2* that may affect leaf shape development [29]. Subsequently, the full-length sequence of *LtuBOP2* was cloned from the transcriptome database of *L. tulipifera,* and its functions were analyzed. BOP2 encodes ANK and a BTB/POZ domain [34], which are widely expressed at the boundary of lateral organs and play a key role in plant morphogenesis [35]; the loss of the *BOP2* function affects the structure of leaves, fruits, and flowers. Using the ClusterX software, the sequences of the BOP2 proteins of *L. tulipifera* and other species were compared. The results showed that these sequences had high homology, with highly conserved BTB and Ank_2 domains. It is speculated that *LtuBOP2* may participate in the initial morphogenesis development of plants, similar to that in other species.

### 3.2. Expression Pattern of LtuBOP2 during Growth and Development

During plant development, the SAM gives rise to leaf primordium and lateral branching primordium at their flanks. These meristem processes are all completed at the lateral organ boundaries, which are restricted growth zones that separate the forming organ and meristem, giving rise to the axillary meristem [36]. Subsequently, the formation of internode and axillary meristems in *Arabidopsis* is inhibited, resulting in the generation of leaves that form a tight rosette. *BOP* has been extensively studied as a boundary defining gene; Norberg et al. performed in situ mapping of *BOP2* mRNA and found that *BOP2* was weakly expressed in early leaf primordium in *Arabidopsis* but more strongly expressed at the site of flower primordium. In our study, *LtuBOP2* expression was highest in stems and relatively high in leaf buds and flower buds, indicating that *BOP2* was specifically expressed in tissues with vigorous meristem activity, which is consistent with Ha et al.’s research [23]. At the later stages of *Arabidopsis* development, *BOP2* expression is restricted to the base of developing leaves [37]. Our study also confirmed the expression specificity of *BOP2* at the base of leaves in *Liriodendron.* The *BOP2*::GUS reporter verified the expression pattern of *BOP2* at the base of mature embryonic cotyledons [38], and our GUS-staining results in *Arabidopsis* showed strong *pLtuBOP2* expression in the petioles at 8 and 12 days after germination (DAG) and in the stem and main veins of leaves at 42 DAG. Taken together, these results indicate that *BOP2* has distinct functions in SAM development and leaf proximal–distal polarity; however, the specific mechanism remains to be investigated.

### 3.3. Roles of LtuBOP2 during Leaf Margin Formation

The meristem originates in the boundary zone. However, in addition to forming the meristem, the boundary also differentiates the proximal parts of the leaflet in the compound leaf and separates individual leaflets from each other [39]. Notably, the boundary zone provides a versatile pattern to maintain morphology establishment. For example, the mechanisms related to leaf primordium initiation also have important functions in leaf development. Furthermore, the axillary and distal leaflet boundaries have pluripotent cell groups that retain the ability to form the meristem [40]. To observe the effects of *LtuBOP2* on plant meristem, we ectopically expressed *LtuBOP2* in *A. thaliana* and obtained a short plant phenotype with apical serration. In previous studies, in the *Arabidopsis* double mutants with *bop1bop2* function loss, the leaves grew transversely along the petiole without obvious petioles, which may be caused by the ectopic growth of the germ cells along the petiole [41]. Overexpression of *BOP1* or *BOP2* led to a reduction in plant height [37], which is consistent with our results. Therefore, the role of *BOP* in leaf development may be to inhibit cell viability.

The process of leaf differentiation includes the formation of vasculature and specialized leaf cells (such as stomata and vessels). The secondary veins of our transgenic plants (35S::*LtuBOP2* and 35S::*LtuBOP2/bop2*) all showed a reduction in varying degrees, indicating that *LtuBOP2* expression resulted in the simplification of the leaf vein architecture system. The leaf blades of the *Osbop1/2/3* triple mutant of *Oryza sativa* L. were shorter, darker, and curlier than those of the wild-type [42]. Defects were observed in the leaf sheath without obvious abnormality at the tip. *OsBOPs* overexpression led to high activity of the mesenchymal tissues and abnormal cell proliferation in the ligules [42]. Generally, boundary cells in *A. thaliana* wild-type often exhibit a characteristic elongated shape [43]. We have observed that the *LtuBOP2* ectopic expression caused boundary cells to disappear at the leaf margin of *A. thaliana* and generated many abnormal epidermal hairs, suggesting that *BOP2* inhibited the formation and development of boundary cells. In tomato, *BOP* inhibits leaflet formation. Therefore, *BOPs* may promote proximal axis development, and regulate the polar differentiation of proximal–distal axis, by inhibiting the differentiation of distal axis differentiation. Notably, *A. thaliana* has lobed leaf edges with the addition of a characteristic shape of protrusions (e.g., serrations) and indentations (e.g., sinuses). Our observation showed that ectopic meristem proliferation was evident at the leaf tip, where serrations were ectopically derived, whereas site growth at the sinuses of leaf margin sinuses was markedly inhibited. Thus, we hypothesized that *LtuBOP2* may act through two different pathways to coordinate the growth and development of serrations at the leaf margin; however, the underlying mechanism is unclear.

### 3.4. BOP2 Affects the Expression of Genes Related to Leaf Development

The lateral organ boundary is a specialized connection separating the initiating lateral organ from the meristem, and the separation of the lateral organ primordium and lateral organ formation is controlled by a complex network of genes [41]. *BP* and *KNAT2* have been implicated in meristem maintenance [44]. *CUCs* are involved in the establishment of SAM by upregulating *STM* and other *KNOX I* [45]. *CUCs* are also repressed in organ primordium by the primordial marker genes *AS1* and *AS2* [46]. *AS1/2* loss of function mutants are defective in the proximal–distal axis but do not affect adaxial–abaxial growth, which is associated with the ectopic expression of the *KNOX* gene (*BP*, *KNAT2,* and *KNAT6*) [47]. Furthermore, Ha et al. reported that there was no change in *BOP* expression in *as1as2* double mutants as well as the reduced expression of *AS2* in *bop1bop2* mutants [23]. Our study also found that *BOP2* overexpression led to *AS2* upregulation, indicating that *BOP* contributes to *AS2* expression during primordial development.

**Figure 8:**
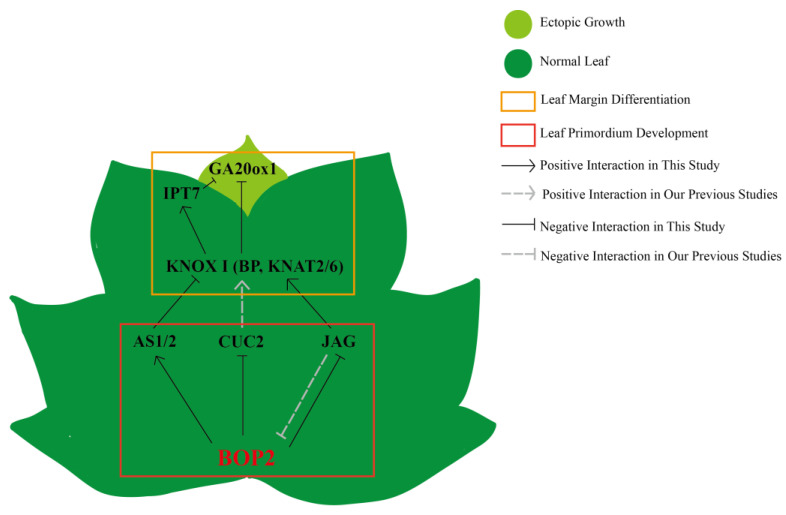
Putative regulation diagram of leaf margin development of *L. tulipifera*. At the initial stage of leaf primordium development, *BOP2* restricted the direction of leaf polarity development by promoting *AS1/2* to inhibit the expression of *CUC2* and *JAG*, and to prevent the excessive proliferation of tissue cells. At the stage of leaf margin differentiation, *BOP2* stimulated the antagonism between *IPT7* and *GA20ox1* by controlling the expression range of *KNOX I* (*BP*, *KNAT2*/*6*) to adjust the degree of leaf margin serration.

Boundary genes, a class of genes specifically expressed in the boundary region between the meristem and leaf primordium, function to define the boundary. Recently, several studies confirmed serration development in single leaves, and that boundary formation between leaflets of compound leaves was inseparable from the action of boundary genes [48]. Leaf morphogenesis goes through several stages, and the location of boundary regions varies among stages. Nevertheless, the boundary zone retains functions that control meristem genes, including the meristem-maintaining special genes *STM*, *BP*, and *CUCs*, which are primarily required for promoting *KNOX I* transcripts to form stem cells in primordium initiation. Notably, these meristem genes are repressed in leaf primordia [23] and flower primordia by *BOP 1/2* [49]. However, *BOP* in turn *1/2* represses *KNOX I* to establish the lateral boundary of the stem organ and leaf polarity during differentiation [50]. This mechanism has been well demonstrated previously. For example, *KNOX I* was ectopically expressed in *bop1bop2* mature leaves [35]. *BP* and *JAG*, which are upregulated in *bop1bop2* [41], therefore promote petiole indeterminate, leading to ectopic leaflet and cotyledon formation [23]. *JAG* ectopic expression suppresses the premature differentiation of tissues, which is necessary for the formation of distal region [21]. Plants with *JAG* ectopic expression were phenotypically similar to the *bop2* mutants, and *AtJAG* was downregulated in the *LtuBOP2*-OE lines. These results confirm that the *BOP2* gene negatively regulates *JAG*. Hormones have long been considered to be the ultimate influencers of serration at the leaf margins. The phytohormone gibberellin (GA), which is thought to antagonize *KNOX* function, is actively degraded by *KNOX I* activity [51]. Moreover, cytokinin (CK) was positively correlated with *KNOX I* expression. Our previous studies confirmed that *JAG* affects the development of apical serrations in leaves by repressing *GA20ox1* and promoting *IPT7* expression. Conversely, we have now demonstrated that *GA20ox1* expression was upregulated, while *IPT7* expression was downregulated in *BOP2*-overexpressing strains, which further confirmed the negative regulatory relationship between *BOP2* and *JAG* (Figure 8). Altogether, we speculate that *BOP2* contributes to normal petioles development and that its regulatory effects may be the partial involvement in the upregulation of the adaxial–abaxial polarity genes *AS1/2* and downregulation of the proximal–distal polarity gene *JAG* during the leaf polarity establishment. More specifically, *BOP2* stimulated the antagonism between *IPT7* and *GA20ox1* by controlling the expression range of *KNOX I* (*BP*, *KNAT2/6*) to repress or regulate hormone content and expression position and to form serrations at leaf margins during leaf morphology development. Although it remains to be determined how phytohormone signaling and gene expression profiles interact with each other, our study provides new insights into the regulatory mechanisms of leaf formation development in *L. tulipifera*.

## 4. Materials and Methods

### 4.1. Plant Materials

*L. tulipifera* and *L. chinense* samples with the same phenological stage were selected as the testing material in the Xiashu Forestry Field (119°13′20″ E, 32°7′8″ N), affiliated with the Nanjing Forestry University (Jurong County, China) [52].

To identify the expression pattern of *BOP2* in various plant tissues at the same time, leaf, flower bud, stem, stamen, pistil, sepal, leaf bud, and petal tissues of *L. tulipifera* and *L. chinense* were sampled in April 2021. To distinguish the expression of the gene at each location in the leaf, three parts of young *L. tulipifera* and *L. chinense* leaves (leaf margin, leaf base, and middle of the leaf) in good condition were collected. To verify *BOP2* expression in leaves at several specific periods, *L. tulipifera* leaves at different developmental stages (the sprouting of leaf bud, immature leaves, mature leaves, and senescence) were collected from April to August 2021. Based on previous studies reporting that the *BOP2* is associated with leaf morphogenesis [23], the leaf polarity establishment, and leaf serration formation, we took leaf buds and leaves from the corresponding stages, including leaf bud germination stage (P1 and P2), immature leaf stage (P3), mature stage (P4 to P6), and senescence stage (P7). Previous studies have reported that the *BOP2* gene is a regulator of lateral organ growth and development [39]; therefore, we categorized the leaves into different parts, namely the leaf margin (a, c, and e), hollow parts of the leaf margin (b and d), petiole (f), and the middle of the leaf (g), to be explored separately.

*A. thaliana* wild-type plants were the Columbia (Col) ecotype. The *A. thaliana bop2* mutant (SALK_075879C) was purchased from the Non-profit Arabidopsis Share Center (FuZhou, China). *A. thaliana* seeds were stored at 4 °C until planting. Before sowing, the seed surfaces were disinfected using 75% alcohol and 10% sodium hypochlorite, germinated on 1/2 Murashige for 10 d, and transferred into the soil. Seedlings were precisely cultivated at 23 °C under a long-day photoperiod (14 h day/10 h night), with 70% relative humidity.

### 4.2. cDNA Synthesis, Sequencing, and Sequence Analysis of LtuBOP2

In terms of the known transcriptome database in other reports [29], EST sequences of the annotated *BOP2* gene were identified in *L. tulipifera*. RACE-cloned primers were created using the Oligo 7 software. RACE was implemented through a SMARTer^®^RACE 5/3 kit (Takara, Beijing, China) and Phanta^®^Max DNA Polymerase (Vazyme Biotech Co. Ltd., Nanjing, China), according to the manufacturer’s instructions for amplifying full-length *LtuBOP2*. Further, they were tested and validated by sequencing (GenScript, Nanjing, China) [53]. Full-length cDNA sequences were obtained through the stitch processing of DNAMAN software (Lynnon Biosoft, San Diego, CA., USA). The open reading frame (ORF) region was predicted by the NCBI ORF Finder (https://www.ncbi.nlm.nih.gov/orffinder/) (accessed on 16 April 2022) to verify the correctness of gene cloning [54]. Protein sequences were aligned from the Cluster W and DNAMAN 8.0 program. Then, a phylogenetic tree was reconstructed with the MEGA7 software, and the Neighbor-Joining (NJ) method based on 1000 bootstrap replicates [55]. The upstream promoter sequence of the *BOP2* gene was found based on the validated CDS sequence. The Plant CARE (http://bioinformatics.psb.ugent.be/webtools/plantcare/html) (accessed on 20 May 2022) was operated to predict the core promoter elements of *pLtuBOP2*.

### 4.3. Recombinant Plasmid Construction and Genetic Transformation

To overexpress the *LtuBOP2* gene in *A. thaliana*, the *LtuBOP2* coding regions were amplified and inserted at the XbaI and BamHI sites of the transformed pBI121 vector downstream of the 35S (Cauliflower mosaic virus) promoter using QuickCut enzymes (Takara, Dalian, China); further, the plasmid 35S::pBI121-*LtuBOP2* was generated using the One Step Cloning Kit (ClonExpress^®^ Ultra Vazyme Biotech Co. Ltd., Nanjing, China). We further imported the recombinant plasmids into *Agrobacterium tumefaciens* (GV3101) and transformed it into *A. thaliana*, applying the floral dipping approach [56]. Kanamycin-resistant primary transformants were gained on 1/2 MS agar plates. Taking the genomic DNA of transgenic leaves in *A. thaliana* as templates, a PCR reaction was accomplished to identify T1 transgenic plants by exploiting specific primers. We acquired stable genetic transgenic plants after the continuous screening of three generations.

### 4.4. RT-qPCR

To explore the effect of *LtuBOP2* on leaf shape formation, we chose some genes that reportedly regulated the leaf serration and designed their primers to verify their transcripts level in 35S::*LtuBOP2* transgenic seedlings, *bop2,* and wild-type. Triplicate seedling samples of the these seedling were prepared at the leaf primordium differentiation stage (10 days after seed germination). Tissue RNA was isolated from various samples of *A. thaliana* and *Liriodendron L* using RNA Extraction Kit (Tiangen, Beijing, China). First-strand cDNA was conflated from 4 µg of total RNA through reverse transcription (PrimeScriptTM RT Master Mix, Takara, Dalian, China) [57]. Quantification of gene expression was performed utilizing a SYBR kit (Takara, Dalian, China) and a 96-hole plate (NEST Biotechnology Co. Ltd., Wuxi, China). *AtACTIN2* from *A. thaliana* and *LcActin97* from *L. chinense* were used as an internal control [58]. Gene expression was calculated using the 2^−∆∆CT^ method with three technical replications and three biological replicates [59].

### 4.5. SEM Observation

To observe cellular changes in the leaf epidermis and leaf serration sites of 35S::*LtuBOP2* and wild-type *A. thaliana* leaves, we sampled the 32-day leaves in an SEM assay. The leaves were placed in a vial, vacuumed with 10 mL of FAA fixative and fixed for 24 h. The fixed material was placed in 50%, 70%, 90%, and 100% ethanol with forceps and dehydrated for 15 min each. The dehydrated material was placed in a 1:2 mixture of isoamyl acetate and alcohol, which was subsequently replaced with 100% isoamyl acetate after 15 min [60]. Then, the samples were treated and observed using SEM, as described by Hepworth et al. [61].

### 4.6. Phenotypic Characterization and Statistical Analysis

The leaves of the homozygous wild-type and *LtuBOP2* transformed plants were photographed by using a digital single-lens reflexes camera (Fujifilm Finepix S5100, Tokyo, Japan) to explore the effect of the *LtuBOP2* overexpression on leaf morphology. The PNG images were taken to calculate the lamina length, lamina width, petiole length, and ratio of lamina length to width of 30 individual leaves through the Digimizer software (www.digimizer.com) (accessed on 17 September 2022) [62]. Statistical analyses were performed using ANOVA Tukey’s test for pairwise comparison. All data are expressed as mean ± standard deviation (SD) of three replicates. Statistical significance was set at *p* < 0.01 and *p* < 0.05, and significantly different values were denoted with different letters.

### 4.7. GUS Histochemical Assay and Venation Pattern Analysis

To identify the expression specificity of *LtuBOP2* in early leaf development, we cloned the *LtuBOP2* promoter sequence, substituted the 35S promoter region with approximately 2 Kb fragment upstream of the translation region of the *LtuBOP2*, and inserted it into the corresponding restriction endonucleatease site of the plasmid pBI121-GUS to form the pBI121-*pLtuBOP2*::GUS structure. The plasmid was then transformed into *A. thaliana*. The seedlings of T2 transgenic positive plants were collected 0, 2, 4, 6, 8 and 12 DAG. To investigate the tissue expression pattern of *LtuBOP2*, we collected cotyledons, young leaves, inflorescences, stems siliques, and seeds 42 days after seed germination at the reproductive stage. Moreover, we used GUS histochemical analysis to detect their GUS activity, and the experimental method was performed as described by Jefferson et al. [63]. High resolution images were taken using a Leica DM500 microscope (Shanghai, China). To compare the mature leaf vein morphology of transformants and mutants, the leaves were directly bleached with 75% (*V/V*) ethanol for 24 h without GUS staining and observed by using a Leica DM500 microscope (Shanghai, China).

## 5. Conclusions

In this study, we cloned and characterized *LtuBOP2*, which is associated with leaf shape development in *L. tulipifera*. Our results confirmed that *LtuBOP2* is a pleiotropic gene, it activated the lateral organ boundary gene *AS1/2* and suppressed the proximal–distal polarity gene *JAG*, and inhibited cell over-differentiation to establish polarity during the establishment of leaf polarity. Furthermore, *BOP2* promotes ectopic meristem formation at the leaf margin by controlling the *KNAT2/6*-mediated upregulation of *GA20ox1* expression during leaf margin expansion, resulting in serrated leaves during the leaf morphology development. Generally, multiple pathways acted in combination to disrupt the balance of endogenous hormones and affect leaf primordium development with subsequent effects on leaf shape. Our study provides evidence for a novel role of *BOP*2 in leaf polarity establishment and provides new insights into leaf shape development in *L. tulipifera*.

## Data Availability

Not applicable.

## Supplementary Materials

The following supporting information can be downloaded at: https://www.mdpi.com/article/10.3390/ijms24043262/s1.
